# Combined effect of anemia and chronic rhinitis on hearing loss in Korean adults: a nationwide observational study

**DOI:** 10.4178/epih.e2024063

**Published:** 2024-07-15

**Authors:** Yeong Jun Ju, Woorim Kim, Jina Han, Soon Young Lee

**Affiliations:** 1 Departement of Preventive Medicine and Public Health, Ajou University School of Medicine, Suwon, Korea; 2 Graduate School of Public Health, Ajou University, Suwon, Korea; 3 National Hospice Center, National Cancer Control Institute, National Cancer Center, Goyang, Korea; 4 Division of Cancer Control & Policy, National Cancer Control Institute, National Cancer Center, Goyang, Korea; 5 Gyeonggi Center for Hypertension and Diabetes, Ajou University, Suwon, Korea

**Keywords:** Hearing loss, Hearing impairment, Anemia, Rhinitis

## Abstract

**OBJECTIVES:**

Studies have suggested an association between hearing loss and anemia. Hearing loss has also been linked to rhinitis, which is characterized by inflammation of the nasal mucous membranes and sinus mucosa. Few studies have concurrently explored the relationships between hearing loss, anemia, and rhinitis. This study was conducted to investigate the association between hearing loss and anemia and to further analyze the potential role of rhinitis in this relationship.

**METHODS:**

Data were collected from the 2020 Korea National Health and Nutrition Examination Survey. Hearing loss was measured with an audiometer in a soundproof booth and was defined as at least moderate impairment (as indicated by a pure-tone average of ≥41 dB in the better-hearing ear). The association between hearing loss and anemia was analyzed using multivariable logistic regression. The combined effect of anemia and rhinitis on hearing loss was assessed with an interaction term.

**RESULTS:**

Among the 2,772 participants, 477 (17.2%) exhibited hearing loss. Participants with anemia were more likely to experience hearing loss than those without anemia (odds ratio [OR], 1.58; 95% confidence interval [CI], 1.07 to 2.33). Furthermore, the odds of hearing loss were greater in participants with both anemia and rhinitis (OR, 3.79; 95% CI, 1.93 to 7.43) relative to those without either condition.

**CONCLUSIONS:**

Anemia was associated with hearing loss in individuals aged 40 years and older. Based on the analysis of combined effects, participants with anemia and chronic rhinitis were more likely to experience hearing loss than individuals without these conditions.

## GRAPHICAL ABSTRACT

[Fig f3-epih-46-e2024063]

## Key Message

Given that anemia is a prevalent and potentially reversible condition, examining this correlation could present novel opportunities for early and effective intervention. This study aimed to evaluate the association between anemia and hearing impairment, while further investigating the role of rhinitis in this relationship. Our findings indicated that individuals with anemia had a higher likelihood of experiencing hearing loss, particularly among those simultaneously affected by chronic rhinitis. Therefore, we emphasize the critical need for effective management of both anemia and rhinitis in addressing public health challenges related to hearing impairment.

## INTRODUCTION

Hearing loss, a common condition in adults, is characterized by the inability to hear sound [1]. The global prevalence of complete hearing loss is estimated at 0.5% in adult males and 0.6% in adult females. Hearing impairment is recognized as a leading cause of disability [2,3]. Various adverse health outcomes have been linked to hearing loss, including diminished quality of life, cognitive decline, and difficulties with activities of daily living [4,5]. As hearing loss is largely preventable, multiple countries currently emphasize the importance of adequate prevention [6,7].

Numerous risk factors for hearing loss have been identified, including age, sex, socioeconomic status, and cardiovascular factors. Nevertheless, uncovering additional risk factors could aid in further preventing and mitigating adverse effects [8]. Iron deficiency anemia has been proposed as another potential risk factor; this condition is relatively prevalent, affecting over one-third of the global population [9]. Anemia is characterized by reduced levels of hemoglobin, a crucial component of red blood cells, in the blood [10]. While the precise mechanism linking hearing loss and anemia remains unclear, one possible explanation is that the blood supply to the inner ear is highly sensitive to ischemic damage [11]. Since anemia is both common and treatable, exploring its relationship with hearing loss could reveal new opportunities for early diagnosis and appropriate management.

In addition to anemia, hearing loss has also been associated with rhinitis, which involves inflammation of the nasal mucous membrane and sinus mucosa [12]. Furthermore, links between anemia and atopic diseases have been reported in multiple cohorts [13]. However, few studies have concurrently explored the relationships between hearing loss, anemia, and rhinitis. Therefore, the present study investigated the association between hearing loss and anemia in adults aged 40 years and older using a large nationwide dataset and further analyzed the potential role of rhinitis in this relationship. The hypothesis was that individuals with anemia would be more likely to experience hearing loss than those without anemia, and this tendency would be stronger in those with both anemia and rhinitis.

## MATERIALS AND METHODS

### Data and study population

**Figure 1.**
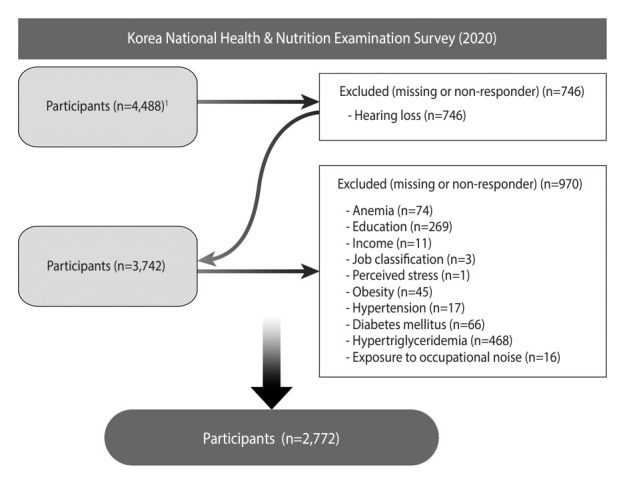
Flowchart illustrating the participant selection process. 1Individuals aged 40 years or above.

This study utilized data from the 2020 Korea National Health and Nutrition Examination Survey (KNHANES). Conducted annually by the Korea Disease Control and Prevention Agency since 1998, the KNHANES is a nationwide cross-sectional survey. It encompasses multistage, stratified area probability samples of civilian non-institutionalized Korean households, categorized by geographic area, age, and sex. The survey comprises 3 components: a health interview, a health examination, and a nutrition survey, all administered by trained medical personnel and dietitians. The present study included individuals aged 40 years and older—the demographic for which the KNHANES assesses hearing loss—for whom complete data were available for the research variables. The final study population comprised 2,772 individuals (Figure 1).

### Outcome measure

The outcome measure in this study was hearing loss, defined as at least a moderate level of hearing impairment. This was indicated by a pure-tone average (PTA) of 41 dB or greater in the better-hearing ear. Such a classification encompasses moderate or more severe forms of hearing loss and aligns with the standard applied in previous studies [14]. Pure-tone audiometric testing was performed using an audiometer (SA 203; Entomed, Malmö, Sweden) inside a soundproof booth, overseen by an otolaryngologist as part of the KNHANES survey. The testing protocol was automated and followed a modified Hughson-Westlake procedure, employing a single, pure tone lasting 1-2 seconds. Frequencies of 0.5 kHz, 1.0 kHz, 2.0 kHz, and 4.0 kHz were tested. A PTA of 41 dB or higher in one or both ears was classified as indicative of hearing loss.

### Independent variables

The independent variable in this study was anemia, which was evaluated through blood sample analysis. Specifically, hemoglobin levels were assessed using the cyanide-free sodium lauryl sulfate method with a hematology analyzer (XE-2100D; Sysmex, Kobe, Japan) [15]. Participants were classified as exhibiting anemia if their hemoglobin levels were below 13 g/dL for male and below 12 g/dL for female, aligning with the standards set by the World Health Organization [15].

The study included a variety of demographic, socioeconomic, and health-related variables as covariates. These variables encompassed rhinitis (categorized as none, seasonal, or chronic), sex (male or female), age group (40-49, 50-59, 60-69, 70-79, or ≥ 80 years), education level (elementary school, middle school, high school, or college), job classification (professional or administrative, office work, sales and service, agriculture and fishery, blue collar, simple labor, or unemployed), current smoking status (no or yes), current drinking status (no or yes, regarding an alcohol consumption frequency of at least once per month), subjective health status (fair or poor), perceived stress (no or yes), obesity (underweight, normal weight, or overweight), hypertension (no, pre-hypertension, or yes), diabetes (no, pre-diabetes, or yes), hypercholesterolemia (no or yes), metabolic syndrome (no or yes), middle ear inflammation (no or yes), abnormal eardrum (no or yes), and occupational noise exposure (no or yes). Rhinitis was assessed through self-reported answers to the question, “In the past 12 months, have you experienced symptoms related to rhinitis, such as sneezing, a runny nose, nasal congestion, or an itchy nose, excluding symptoms from a cold?” Respondents could answer “yes” or “no.” Those who answered “yes” were further questioned to determine whether their symptoms were seasonal or persistent throughout the year. Obesity was determined based on body mass index (BMI), with a BMI of less than 18.5 kg/m^2^ indicating underweight, 18.5 kg/m^2^ to 24.9 kg/m^2^ representing normal weight, and 25.0 kg/m^2^ or above signifying obesity. Hypertension was classified based on blood pressure levels, with a systolic blood pressure (SBP) of 140 mmHg or higher and/or a diastolic blood pressure (DBP) of 90 mmHg or higher indicating hypertension. An SBP between 120 mmHg to 139 mmHg and/or a DBP between 80 mmHg to 89 mmHg were considered indicative of pre-hypertension. Middle ear inflammation was identified based on a physician’s diagnosis. Abnormal eardrum was determined through tympanometry test results. Diabetes was defined as having a fasting blood sugar level of 126 mg/dL or higher, using oral hypoglycemic agents or insulin, having been diagnosed with diabetes by a physician, or having a glycosylated hemoglobin (A1C) level of 6.5% or higher. Pre-diabetes was indicated by a fasting blood sugar level of 100 mg/dL to 125 mg/dL or an A1C level of 5.7% to 6.4%. Hypercholesterolemia was defined as having a fasting total cholesterol level of at least 240 mg/dL or taking lipid-lowering medication. Hypertriglyceridemia was identified in participants with a fasting triglyceride level of 200 mg/dL or higher.

### Statistical analysis

The chi-square test was used to assess differences between groups and to examine the general characteristics of the participants. Multivariable logistic regression analysis was conducted to explore the relationship between hearing loss and anemia. The combined effect of anemia and chronic rhinitis on hearing loss was assessed using an interaction term. Before these variables were combined, the interaction between them was analyzed to assess the appropriateness of their combination. The result of this test was statistically significant; thus, the combined effect of anemia and chronic rhinitis on hearing loss was analyzed. This analysis was adjusted for numerous covariates, including sex, age, education level, job classification, smoking status, drinking status, subjective health status, perceived stress, obesity, hypertension, diabetes, hypercholesterolemia, metabolic syndrome, middle ear inflammation, abnormal eardrum, and occupational noise exposure. The results were expressed as odds ratios (ORs) with 95% confidence intervals (CIs). The p-values were 2-sided, and a p-value of less than 0.05 was considered to indicate statistical significance. All statistical analyses were performed using SAS version 9.4 (SAS Institute Inc., Cary, NC, USA).

### Ethics statement

This study was conducted in accordance with the Declaration of Helsinki and its subsequent amendments. The KNHANES data are open-access, and all personal information was fully anonymized prior to its release. As the data were exempt from institutional review board review, this study was excluded from the review list pursuant to Article 2.2 of the Enforcement Rule of the Bioethics and Safety Act in Korea.

## RESULTS

The general characteristics of the participants are presented in Table 1. The study comprised 2,772 adults aged 40 and older, with hearing loss identified in 477 (17.2%) of these individuals. The prevalence of hearing loss increased with age and was higher among individuals with anemia (28.4%) than in those without anemia (15.6%). Additionally, hearing loss was more common among participants with chronic rhinitis (23.3%) compared to those with seasonal rhinitis (12.3%) or no rhinitis (16.9%).

**Figure 2.**
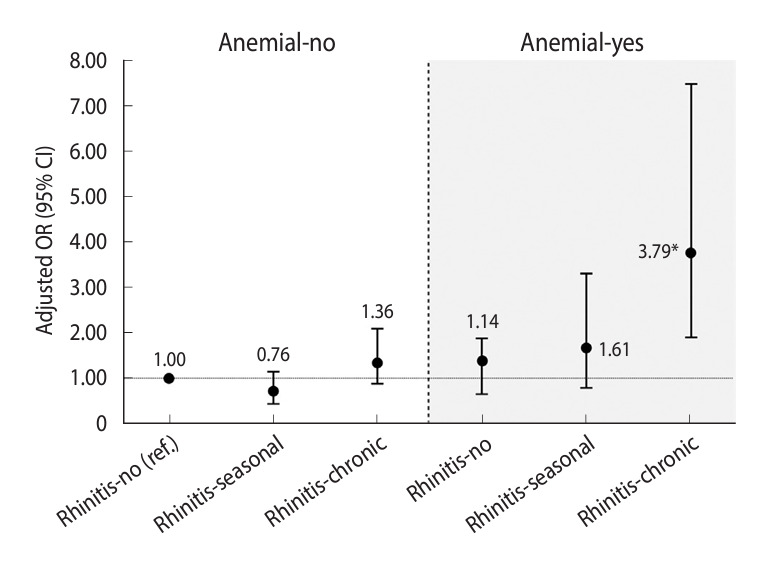
Combined effect of anemia and rhinitis on hearing loss. Adjusted for all covariates. OR, odds ratio; CI, confidence interval. ^*^p<0.05.

The results of the multivariable logistic regression analysis examining the association between hearing loss and anemia are presented in Table 2. Participants with anemia were more likely to experience hearing loss than those without anemia (OR, 1.58; 95% CI, 1.07 to 2.33). Individuals with chronic rhinitis displayed increased odds of hearing loss (OR, 1.60; 95% CI, 1.14 to 2.24) relative to those without rhinitis. Figure 2 illustrates the combined effect of anemia and rhinitis on hearing loss. A significantly higher risk of hearing loss was observed among participants with both anemia and chronic rhinitis (OR, 3.79; 95% CI, 1.93 to 7.43) compared to those with neither condition (p for interaction=0.033).

## DISCUSSION

This study investigated the association between hearing loss and anemia in adults aged 40 years and older, utilizing nationally representative cross-sectional data from Korea. Additionally, it examined the role of rhinitis in this association. Hearing loss was defined as at least moderate impairment, as indicated by a PTA of 41 dB or higher in the better-hearing ear. Approximately 17% of participants exhibited hearing loss, which was more prevalent among those with anemia than in those without. Likewise, individuals with anemia displayed significantly higher odds of experiencing hearing loss compared to participants without anemia. In the combined effects model, individuals with both anemia and chronic rhinitis had the highest likelihood of exhibiting hearing loss, significantly higher than those with neither condition.

The mechanisms by which anemia may increase the risk of hearing loss remain unclear, although the literature suggests a potential link between anemia and sensorineural hearing loss. Research indicates that the vascular and neurological effects of iron deficiency anemia, which accounts for approximately half of the global anemia burden, may be linked to cochlear sensitivity [16]. The cochlea is particularly susceptible to reduced blood oxygen levels caused by iron deficiency anemia, as it receives blood exclusively through the labyrinthine artery [17]. Consequently, the lack of collateral circulation associated with iron deficiency anemia may render the cochlea prone to ischemia [11]. Additionally, iron plays a key role in the neurological system; thus, anemia could relate to the development of various neurological disorders, such as epilepsy and dementia, which may also be linked to hearing impairment [16,18,19].

Apart from anemia, this study also revealed a statistically significant combined effect of anemia and rhinitis on hearing loss. Individuals with both anemia and chronic rhinitis had higher odds of experiencing hearing loss, with a significant interaction. This trend may stem from the previously established fact that hearing loss is independently associated with rhinitis. Severe rhinitis can lead to complications such as sinusitis or Eustachian tube dysfunction, which in turn can cause conductive hearing loss [20]. The physiological relationship between the nose and ear may increase the risk of conductive hearing loss [21]. Additionally, rhinitis has been implicated in sensorineural hearing loss, as inflammation from histamine can impact inner ear function [20]. This relationship is supported by previous studies, which suggest that inflammatory mediators and toxic byproducts from the antibody response can disrupt the function of hair cells in the ear [22]. Moreover, anemia and iron deficiency have been linked to allergic diseases, including allergic rhinitis [23]. Chronic inflammation can affect the immune system’s regulation of iron homeostasis, leading to anemia [24]. Given the independent associations between hearing loss and both anemia and rhinitis, as well as the elevated likelihood of anemia in individuals with allergic rhinitis, it is reasonable that the co-occurrence of anemia and rhinitis demonstrated a stronger connection to the risk of hearing loss in this study.

This study has several limitations. First, the KNHANES data are cross-sectional, which precludes making causal inferences from the study results. Due to the nature of the data, an extended history of chronic rhinitis was not recorded; therefore, the long-term associations between the variables within the pathology of hearing loss could not be fully explored. Future longitudinal studies are necessary to elucidate the directionality of the relationship under investigation. Second, due to data constraints, this study could not differentiate between sensorineural and conductive hearing loss. The dataset does not include information on additional tests performed to distinguish conductive hearing loss, which is estimated to account for less than 10% of hearing loss in adults [25]. Nevertheless, the KNHANES data have been extensively utilized to measure and report hearing loss in prior studies [26]. Since the underlying mechanisms of sensorineural and conductive hearing loss differ, further research that distinguishes between these types is essential for a more in-depth understanding of the topic. Third, the measurement and categorization of rhinitis were based on self-reports. While the question used to assess rhinitis was carefully worded to exclude symptoms from a common cold, the potential for recall bias cannot be eliminated. Other health-related variables were measured using objective health examinations and laboratory results, but this methodology limited the analysis of any potential dose-response relationship of the combined effect of rhinitis and anemia on hearing loss. Fourth, although hearing loss was measured using objective health examinations designed to identify moderate or more severe impairment, the degree of hearing loss could not be quantified due to data limitations. Lastly, the study population was limited to individuals aged 40 years and older, as younger Koreans were not assessed for hearing loss in the KNHANES. Consequently, generalizing the study results to all age groups is challenging.

Despite these limitations, this study stands out for its investigation into the link between anemia and hearing loss using large, nationally representative data. Furthermore, it explored the combined impact of anemia and rhinitis on hearing loss. Given the repeated suggestions from diverse studies regarding a connection between anemia and hearing loss, this research aimed to examine this potential correlation in Korea using existing survey data. By identifying a significant association, the study paves the way for further research to establish a causal relationship between these conditions, which is crucial as hearing loss remains a global health concern that is largely preventable and treatable. Future studies must examine the effectiveness of screening for hearing loss in patients with anemia or rhinitis, who may be at increased risk. It is important to consider strategies for incorporating hearing loss screening into clinical practice, which could reduce the public health burden of hearing loss through prevention and early intervention.

In conclusion, anemia was associated with a higher likelihood of hearing loss in individuals aged 40 and older. When analyzing the combined effect of anemia and rhinitis on hearing loss, individuals with both anemia and chronic rhinitis were more likely to experience hearing loss compared to those without either condition. These findings highlight the importance of managing anemia and rhinitis in the context of public health strategies addressing hearing loss. Further research is necessary to more clearly elucidate the relationships between hearing loss, anemia, and rhinitis.

## Figures and Tables

**Figure f3-epih-46-e2024063:**
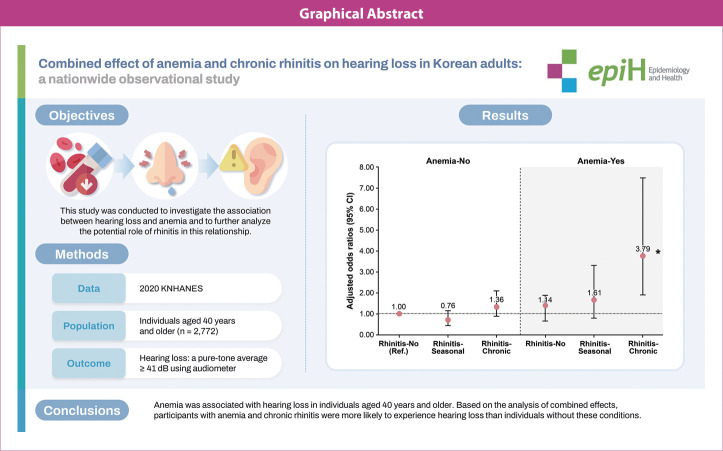


**Table 1.** General characteristics of study participants

Characteristics	Total	Hearing loss		p-value
		No	Yes	
Anemia				<0.001
No	2,420 (87.3)	2,043 (84.4)	377 (15.6)	
Yes	352 (12.7)	252 (71.6)	100 (28.4)	
Rhinitis				<0.001
None	1,665 (60.1)	1,383 (83.1)	282 (16.9)	
Seasonal	571 (20.6)	501 (87.7)	70 (12.3)	
Chronic	536 (19.3)	411 (76.7)	125 (23.3)	
Sex				<0.001
Male	1,197 (43.2)	923 (77.1)	274 (22.9)	
Female	1,575 (56.8)	1,372 (87.1)	203 (12.9)	
Age (yr)				<0.001
40-49	638 (23.0)	629 (98.6)	9 (1.4)	
50-59	715 (25.8)	671 (93.9)	44 (6.1)	
60-69	752 (27.1)	632 (84.0)	120 (16.0)	
≥70	667 (24.1)	363 (54.4)	304 (45.6)	
Education level				<0.001
Elementary school	637 (23.0)	409 (64.2)	228 (35.8)	
Middle school	348 (12.5)	280 (80.5)	68 (19.5)	
High school	894 (32.3)	773 (86.5)	121 (13.5)	
College	893 (32.2)	833 (93.3)	60 (6.7)	
Income				0.039
Q1	642 (23.2)	512 (79.8)	130 (25.2)	
Q2	692 (25.0)	567 (81.9)	125 (18.1)	
Q3	700 (25.2)	586 (83.7)	114 (16.3)	
Q4	738 (26.6)	630 (85.4)	108 (14.6)	
Job classification				<0.001
Professional or administrative	346 (12.5)	329 (95.1)	17 (4.9)	
Office work	234 (8.4)	224 (95.7)	10 (4.3)	
Sales and service	326 (11.8)	299 (91.7)	27 (8.3)	
Agriculture and fishery	132 (4.7)	94 (71.2)	38 (28.8)	
Blue-collar	249 (9.0)	210 (84.3)	39 (15.7)	
Simple labor	291 (10.5)	227 (78.0)	64 (22.0)	
Unemployed	1,194 (43.1)	912 (76.4)	282 (23.6)	
Current smoking				0.732
No	2,379 (85.8)	1,972 (82.9)	407 (17.1)	
Yes	393 (14.2)	323 (82.2)	70 (17.8)	
Current drinking (at least once per month)				<0.001
No	1,506 (54.3)	1,194 (79.3)	312 (20.7)	
Yes	1,266 (45.7)	1,101 (87.0)	165 (13.0)	
Subjective health status				<0.001
Fair	758 (27.3)	660 (87.1)	98 (12.9)	
Poor	2,014 (72.7)	1,635 (81.2)	379 (18.8)	
Perceived stress				0.002
No	2,139 (77.2)	1,745 (81.6)	394 (18.4)	
Yes	633 (22.8)	550 (86.9)	83 (13.1)	
Obesity (kg/m^2^)				0.051
Underweight (BMI <18.5)	60 (2.2)	52 (86.7)	8 (13.3)	
Normal (18.5≤BMI<25.0)	1,624 (58.6)	1,321 (81.3)	303 (18.7)	
Obese (BMI ≥25.0)	1,088 (39.2)	922 (84.7)	166 (15.3)	
Hypertension				<0.001
No	903 (32.6)	815 (90.3)	88 (9.7)	
Pre-hypertension	721 (26.0)	608 (84.3)	113 (15.7)	
Yes	1,148 (41.4)	872 (76.0)	276 (24.0)	
Diabetes				<0.001
No	883 (31.8)	773 (87.5)	110 (12.5)	
Pre-diabetes	1,318 (47.6)	1,091 (82.8)	227 (17.2)	
Yes	571 (20.6)	431 (75.5)	140 (24.5)	
Hypertriglyceridemia				0.706
No	2,385 (86.0)	1,972 (82.7)	413 (17.3)	
Yes	387 (14.0)	323 (83.5)	64 (16.5)	
Hypercholesterolemia				0.299
No	1,835 (66.2)	1,529 (83.3)	306 (16.7)	
Yes	937 (33.8)	766 (81.8)	171 (18.2)	
Middle ear inflammation				<0.001
No	2,632 (95.0)	2,204 (83.7)	428 (16.3)	
Yes	140 (5.0)	91 (65.0)	49 (35.0)	
Abnormal eardrum				<0.001
No	2,581 (93.1)	2,183 (84.6)	398 (15.4)	
Yes	191 (6.9)	112 (58.6)	79 (41.4)	
Occupational noise exposure				<0.001
No	2,325 (83.9)	1,955 (84.1)	370 (15.9)	
Yes	447 (16.1)	340 (76.1)	107 (23.9)	
Total	2,772 (100)	2,295 (82.8)	477 (17.2)	

Values are presented as number (%). Q, quartile; BMI, body mass index.

**Table 2.** Association between hearing loss and anemia^1^

Variables	Hearing loss
Anemia	
No	1.00 (reference)
Yes	1.58 (1.07, 2.33)
Rhinitis	
None	1.00 (reference)
Seasonal	0.84 (0.57, 1.22)
Chronic	1.60 (1.14, 2.24)
Sex	
Male	1.00 (reference)
Female	0.37 (0.26, 0.52)
Age (yr)	
≥70	1.00 (reference)
40-49	0.02 (0.01, 0.06)
50-59	0.11 (0.07, 0.19)
60-69	0.22 (0.16, 0.32)
Education level	
Elementary school	1.00 (reference)
Middle school	0.65 (0.42, 1.00)
High school	0.78 (0.54, 1.14)
College	0.43 (0.25, 0.75)
Income	
Q1	1.00 (reference)
Q2	1.10 (0.75, 1.63)
Q3	0.75 (0.50, 1.12)
Q4	0.93 (0.63, 1.39)
Job classification	
Professional or administrative	1.00 (reference)
Office work	0.47 (0.18, 1.23)
Sales and service	0.84 (0.37, 1.88)
Agriculture and fishery	1.02 (0.44, 2.39)
Blue-collar	1.31 (0.58, 2.96)
Simple labor	1.11 (0.51, 2.39)
Unemployed	1.04 (0.53, 2.07)
Current smoking	
No	1.00 (reference)
Yes	1.11 (0.71, 1.72)
Current drinking (at least once per month)	
No	1.00 (reference)
Yes	0.72 (0.53, 0.98)
Subjective health status	
Fair	1.00 (reference)
Poor	1.04 (0.74, 1.47)
Perceived stress	
No	1.00 (reference)
Yes	1.41 (0.96, 2.06)
Obesity (kg/m^2^)	
Underweight (BMI <18.5)	1.00 (reference)
Normal (18.5≤BMI<25.0)	0.97 (0.36, 2.60)
Obese (BMI ≥25.0)	0.78 (0.28, 2.12)
Hypertension	
No	1.00 (reference)
Pre-hypertension	0.89 (0.59, 1.36)
Yes	1.08 (0.75, 1.57)
Diabetes	
No	1.00 (reference)
Pre-diabetes	1.06 (0.75, 1.50)
Yes	0.98 (0.67, 1.44)
Hypertriglyceridemia	
No	1.00 (reference)
Yes	1.61 (1.05, 2.46)
Hypercholesterolemia	
No	1.00 (reference)
Yes	1.03 (0.75, 1.41)
Middle ear inflammation	
No	1.00 (reference)
Yes	3.46 (2.13, 5.62)
Abnormal eardrum	
No	1.00 (reference)
Yes	2.46 (1.61, 3.77)
Occupational noise exposure	
No	1.00 (reference)
Yes	1.36 (0.94, 1.95)

Values are presented as adjusted odds ratio (95% confidence interval). Q, quartile; BMI, body mass index.

1 Adjusted for rhinitis, sex, age, education level, income, job classification, current smoking status, current drinking status, subjective health status, perceived stress, obesity, hypertension, diabetes, hypercholesterolemia, hypertriglyceridemia, and occupational noise exposure.
